# Congenital Fungus Hæmatodes

**Published:** 1840-07

**Authors:** 


CONGENITAL FUNGUS HÆMATODES.

THE first and only case of congenital fungus hæmatodes, or Encephaloid, we have ever seen, fell under our observation, in the vicinity of Athens, Ohio, in a late trip to that town. We were indebted to Drs. Carpenter and Blackstone for the opportunity of seeing it.

The child, a female, was two days old. The disease occupies the whole of the left nates, which is swelled out into a great, shining, globoid tumour; rendered irregular by tuberous elevations. It extends from the perineum, anus and vulva to the sacrum and the spine of the ilium, so as to involve the acetabulum. Its colour is deep red. Many of the cutaneous veins are much enlarged. A portion of the skin had suffered abrasion and considerable hæmorrhage had already occurred. Some parts of the tumour are hard, others soft — the general mass felt hard. It was tender under the hand. In short it had all the diagnostic marks of encephaloid, before fungous growths appear. The child was rather lean but seemed to be in health; and was free from tumors or malformations in any other part. From this case it would appear, that this variety of carcinoma occurs throughout a much more extended period of life than schirrhus, for we have seen it up to the 50th year — while the latter prevails most in advanced life, is perhaps never congenital and not often a complaint of youth. D.

